# Physicochemical Study on the Strength Development Characteristics of Cold Weather Concrete Using a Nitrite–Nitrate Based Accelerator

**DOI:** 10.3390/ma12172706

**Published:** 2019-08-23

**Authors:** Heesup Choi, Masumi Inoue, Hyeonggil Choi, Jihoon Kim, Yuhji Sudoh, Sukmin Kwon, Bokyeong Lee, Akira Yoneyama

**Affiliations:** 1 Department of Civil and Environmental Engineering, Kitami Institute of Technology, Hokkaido 090-8507, Japan; 2 School of Architecture, Kyungpook National University, Daegu 41566, Korea; 3 Faculty of Environmental Technology, Muroran Institute of Technology, Hokkaido 090-8585, Japan; 4 Basic Chemicals Department Chemicals Division, Nissan Chemical Corporation, Tokyo 103-6119, Japan; 5 Department of Construction Technology Research, Land & Housing Institute, Daejeon 34047, Korea; 6 Intelligent Construction Automation Center, Kyungpook National University, Daegu 41566, Korea

**Keywords:** Cold weather concreting, anti-freezing agent, nitrite–nitrate based accelerator, NO_2_^−^, NO_3_^−^, C_3_A, ettringite, strength

## Abstract

There has recently been an increased use of anti-freezing agents that are primarily composed of salt- and alkali-free calcium nitrite (Ca(NO_2_)_2_) and calcium nitrate (Ca(NO_3_)_2_) to promote the hydration reaction of concrete in cold weather concreting. Nitrite–nitrate based accelerators accelerate the hydration of C_3_A and C_3_S in cement more quickly when their quantities are increased, thereby boosting the concrete’s early strength and effectively preventing early frost damage. However, the connection between the hydrate formation behavior and the strength development characteristic over time has yet to be clearly identified. Therefore, in this study, a wide range of physicochemical reviews were carried out to clarify the relationship between the hydrate formation behavior and the strength development characteristics, both at an early age and at later ages, which results from the addition of nitrite–nitrate based accelerators to concrete in varying amounts. These accelerators also act as anti-freezing agents. The results show that an increased quantity of nitrite–nitrate based accelerators caused an increase in the early strength of the concrete. This was due to the formation of nitrite and nitrate hydrates in large amounts, in addition to ettringite containing SO_4_^2^, which is generated during the hydration reaction of normal Portland cement at an early age. On the other hand, at later ages, there was a rise in nitrite and nitrate hydrates with needle crystal structures exhibiting brittle fracture behavior. A decrease in C–S–H gel and Ca(OH)_2_ hydrates, deemed to have caused a decline in strength on Day 3 and thereafter, was also observed.

## 1. Introduction

In cold weather concreting, early frost damage caused by water freezing in the concrete during the early stages of condensation and hardening may cause a serious decline in performance of the concrete with regard to its quality, strength, and durability [1,2]. As a measure to prevent early frost damage, an early-stage curing temperature condition has been set forth in Japan, aiming to prevent concrete from freezing before its early strength reaches 5 N/mm^2^ [3]. In this measure, when delays in concrete strength development are expected at a cold weather concreting site, the mixing ratio is corrected and insulation and heat curing are performed to mitigate the issue [4,5]. A common method of heat curing in cold weather concreting is to cover the area around the concrete structure in the concreting layer with an insulating waterproof cloth and to heat the area under the cloth using a hot air blower (jet heater) [4,5]. This procedure, however, has low thermal efficiency, a risk of fire, and generates combustion gases (CO, CO_2_, etc.). Thus, according to prior studies [4,5,6], it may have an adverse impact on the strength development durability of the concrete structure. 

There are also cases in which heat curing by heater or a temporary tent is difficult due to a steep slope, restricted and narrow workplaces, rainstorms, snowstorms, or other factors [7]. In these types of environments, there has been a gradual increase in the use of anti-freezing agents that are nearly unaffected by storm water and rainstorms. These agents can effectively prevent early frost damage and promote the hydration reaction in combination with curing sheets by acting as an admixture that promotes hardening of the concrete [8]. The allowable ambient temperature for which anti-freezing agents can prevent early frost damage in cold weather concreting is dependent on the member dimensions, curing conditions, concrete temperature during placement, and concrete mixing ratios. In the case of Japan, for instance, the use of anti-freezing agents is permitted in the foundation of housing structures with a member thickness of no more than 15 cm. Their use is further restricted to cases where the ambient temperature ranges from −4 °C to −8 °C [3,7,8]. However, there have not been any discussions regarding regulations for the use of anti-freezing agents under extremely cold conditions, when the temperature drops to −10 °C or lower. In many of the cold regions of the world, including Hokkaido, Japan, cold weather concreting at temperatures below −10 °C is unavoidable. In order to boost the effectiveness of early frost damage prevention through the promotion of the hydration reactions, there is a need to adjust and increase the concentration of the anti-freezing agents. 

Further, the vast majority of the anti-freezing agents used in cold weather concreting in Japan have salt-free, alkali-free calcium nitrite (Ca(NO_2_)_2_) or calcium nitrate (Ca(NO_3_)_2_) as the primary ingredients [7,8]. An adequate amount of these agents is added to the concrete to promote the reaction between NO_2_^−^ and NO_3_^−^ ions in the nitrite–nitrate based accelerator with the Al_2_O_3_ of C_3_A in the concrete. This, in turn, increases the amount of nitrite or nitrate hydrates [9], thus promoting the early strength of concrete, according to existing studies [2,6,10]. However, when an excess amount of nitrite–nitrate based accelerator is added, it leads to the production of needle-type SO_4_^2^ ettringite (Aft; 3CaO·Al_2_O_3_·3CaSO_4_·32H_2_O) which is generated in the hydration reaction of normal Portland cement. Further, nitrite hydrates (nitrite-Aft; 3CaO·Al_2_O_3_·Ca(NO_2_)_2_·10H_2_O) and nitrate hydrates (nitrate-AFt; 3CaO·Al_2_O_3_·3Ca(NO_3_)_2_·16-18H_2_O) with a similar needle-type crystal structure are also generated in large amounts. This may result in an adverse effect on the strength development at mid and late ages [9,10]. So far, there have been many studies on the effects of increasing the amount of nitrite–nitrate based accelerators on early strength development in low-temperature environments. However, there are few studies investigating the correlation between the strength development characteristic of concrete at mid and late ages and the amount of hydration products formed, which result from the addition of a large amount of a nitrite–nitrate based accelerator.

**Figure 1:**
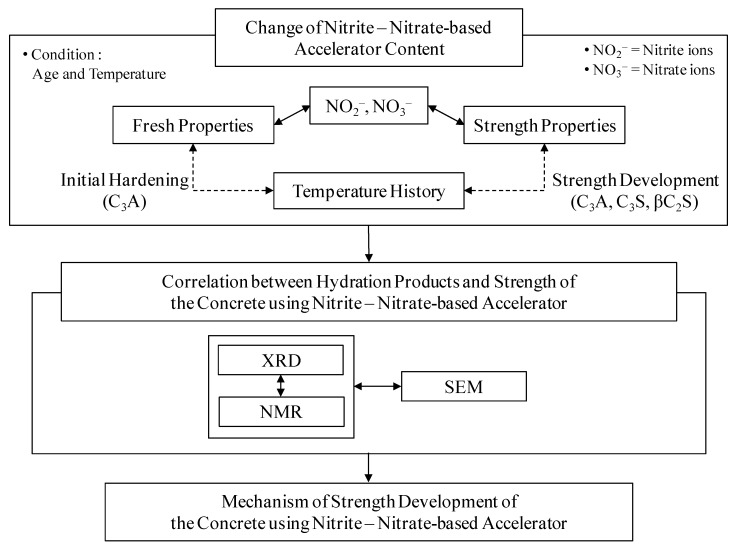
Study flow chart.

Therefore, in this study, diverse physicochemical reviews were performed in order to clarify the relationship between the hydrate formation behavior and the strength development characteristic at the early age and later ages. These hydrate formations are the result of adding nitrite–nitrate based accelerators, which are anti-freezing agents, to concrete in varying amounts. Specifically, a physical experiment was carried out on a concrete mixture where the quantity of the nitrite–nitrate based accelerator was correlated with fluidity, compressive strength, and changes in internal temperature across time. Microanalyses using X-ray diffraction (XRD), nuclear magnetic resonance (NMR), and scanning electronic microscope (SEM) were carried out for quantitative comparative analyses of the strength development characteristics. Moreover, these analyses increased the understanding of the formation behavior of the hydrates. Figure 1 shows the flow chart for this study.

## 2. Experimental Overview

### 2.1. Materials and Methods

A large amount of a nitrite–nitrate based accelerator results in a more rapid reaction between the nitrite ions (NO_2_^−^) and nitrate ions (NO_3_^−^) and Al_2_O_3_ of C_3_A in the cement which promotes the condensation and hardening of concrete immediately after mixing [9]. Accordingly, in this study, concentrations of NO_2_^−^ from Ca(NO_2_)_2_ and NO_3_^−^ from Ca(NO_3_)_2_, which are the main components of the nitrite–nitrate based accelerator (CN), were adjusted in an aqueous solution to a proportional ratio of 45%, as shown in Table 2. Table 1 shows the properties of the materials used, Table 2 shows the properties of the anti-freezing agent used in this study, and Table 3 shows the mixing ratio of the mortar used in this experiment. The water-to-cement ratio was set at 50%, based on the Operation Manual of Anti-freezing agents in Japan, and S/C was set to be 2.0 [7,8]. Further, the standard amount of nitrite–nitrate based accelerator (CN), used as an anti-freezing agent in cold weather concreting, is around 4–7% (3–5 L per 100 kg of cement) of the cement mass [8]. Therefore, an addition of CN at 7% or more was defined as a “large quantity” and the experiment was carried out with CN added at five different ratios: 0%, 7%, 9%, 11%, and 13%.

### 2.2. Experimental Method

The Operation Manual of Anti-freezing Agents implemented in Japan prescribes the need to maintain the internal temperature of concrete at a minimum of 5 °C for a minimum of 24 h after mixing if a nitrite–nitrate based accelerator (CN) is added to concrete as an accelerating admixture [8]. Moreover, in the “Recommendation for practice of cold weather concreting” issued by the Architectural Institute of Japan, it is prescribed that the mixing and placement of concrete be performed within a temperature range of +10 to +20 °C [3]. Therefore, in this experiment, the materials were maintained and the mortar specimens were fabricated at 10.0 ± 1 °C and 85 ± 5% RH for identifying the hydration mechanism when a large amount of a nitrite–nitrate based accelerator (CN) was added as an anti-freezing agent. Next, sealed curing was performed under the aforementioned temperature and humidity conditions, and various physicochemical assessments were conducted at various ages using the experimental method outlined below. It should be noted, however, that in this study, “early age” was defined as “within 24 h after mixing” and “mid and late ages” were defined as the time period after that initial 24-hour time span. Table 4 shows the conditions and assessment method applied in this experiment.

As for the concrete’s fresh state characteristic, a flow experiment was carried out immediately after mixing the mortar, in accordance with “Flow test” in JIS R 5201 (Tokyo, Japan) [11]. 

To assess the compressive strength, sealed curing was performed at 10.0 ± 1 °C and 85% ± 5% RH from Day 1 to Day 14 after placing the mortar into a φ 5×10 cm mold. Next, the compressive strength was measured at each age (Days 1, 3, 7, and 14). 

In the case of internal temperature, the changes in the temperature at the center of the mortar were measured over time, from immediately after mixing until Day 14, by installing a thermo couple at the center of the φ 10 × 20 cm mold. 

XRD was performed using a Rigaku Smart LabX X-Ray Diffractometer (Tokyo, Japan) under the following conditions: a tube voltage of 40 kV, tube current of 20 mA, scanning range of 2θ = 5–65°, scan step of 0.02°, and scan speed of 1°/min. In addition, 5 wt% alpha-alumina (α-Al_2_O_3_) was used as the internal standard substance. 

^27^Al MAS NMR measurements were performed at an observation frequency of 208.6 MHz using an ECA-800 (18.8T) NMR Spectrometer (Tokyo, Japan). Furthermore, a sample tube with a diameter of 3.8 mm was used to perform the analysis at a spinning speed of 20 kHz, pulse width of 0.9 μs, and a relaxation delay of 0.5 s for 1,280 scans. All NMR data were used to determine the deconvolution and peak area from the Lorentzian function using Delta Software. 

**Figure 2:**
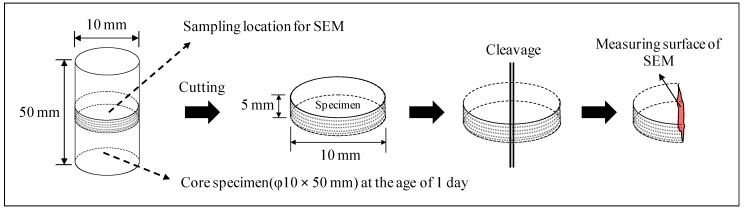
Sampling of specimens for SEM.

As for SEM observations, the JSM-6380 Scanning Electron Microscope (Tokyo, Japan) was used to examine the samples at 1,000× and 5,000× magnifications at a voltage of 15 kV. The samples used in the SEM observations were coated with platinum. SEM observations were obtained from the cleavage plane (inside) of specimens, as shown in Figure 2.

The samples used in the microanalyses (XRD, NMR, SEM) of this study were of various ages that had been immersed in acetone for at least four hours to stop the hydration reaction.

## 3. Results and Discussion

### 3.1. Fresh State Characteristics

**Figure 3:**
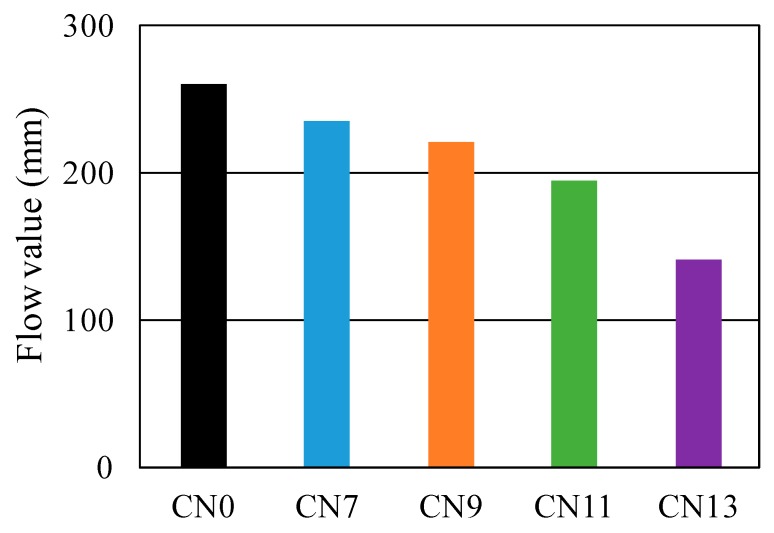
Change of flow value.

**Figure 4:**
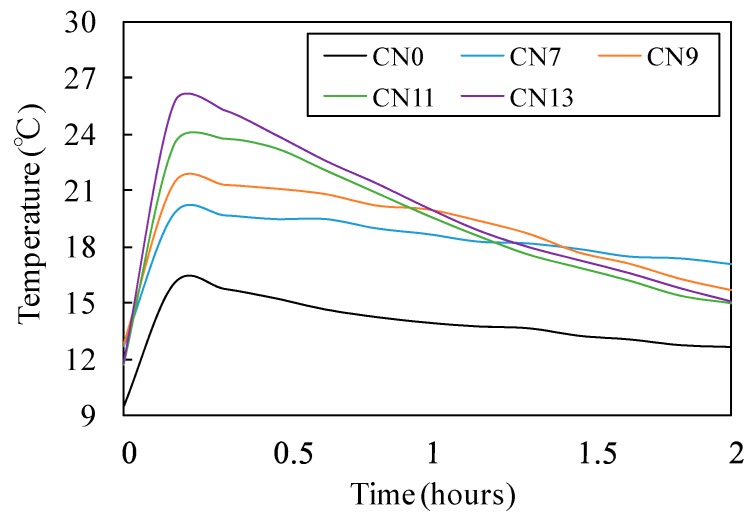
Temperature history (2 h).

Figure 3 shows the results of the flow test in each case with a different amount of the nitrite–nitrate based accelerator (CN) added, while Figure 4 shows the changes in the internal temperature of each mortar specimen from immediately after the mixing process until two hours afterwards. 

Flow results shown in Figure 3 are 260 mm for CN0, 240 mm for CN7, 220 mm for CN9, 190 mm for CN11, and 140 mm for CN13. These finding suggest that fluidity tends to decline as the amount of CN increases. It is noteworthy that the flow value of CN0, to which CN was not added, was 260 mm, while the flow value of CN13, with 13% CN, was 140 mm, indicating an approximate 46% reduction in fluidity. The highest internal temperatures of the mortar specimens immediately after the mixing process were, as shown in Figure 4, 16.1 °C for CN0, 19.8 °C for CN7, 21.4 °C for CN9, 23.8 °C for CN11, and 25.7 °C for CN13, indicating a positive correlation between the amount of CN added and the rate at which the internal temperature rose after the mixing process. In particular, the internal temperature of CN13 increased to 25.7 °C, approximately 10 °C higher than the internal temperature of CN0, which was 16.1 °C. 

When CN is added to concrete as an accelerator, it leads to a rapid reaction between the nitrite (NO_2_^−^) and nitrate ions (NO_3_^−^) from the CN and Al_2_O_3_ of C_3_A in the cement. In addition, the hydration reaction of normal Portland cement produces SO_4_^2−^ ettringite (AFt). This, in turn, results in the production of large amounts of nitrite hydrates (nitrite-Aft; 3CaO·Al_2_O_3_·Ca(NO_2_)_2_·10H_2_O) and nitrate hydrates (nitrate-Aft; 3CaO·Al_2_O_3_·3Ca(NO_3_)_2_·16-18H_2_O) [9,10]. Therefore, as an increasing amount of CN is added, the heat of hydration generated from the production of nitrite and nitrate hydrates increases, and this causes the raise in internal temperature of the mortar as well as reduced fluidity.

### 3.2. Compressive Strength Characteristics

**Figure 5:**
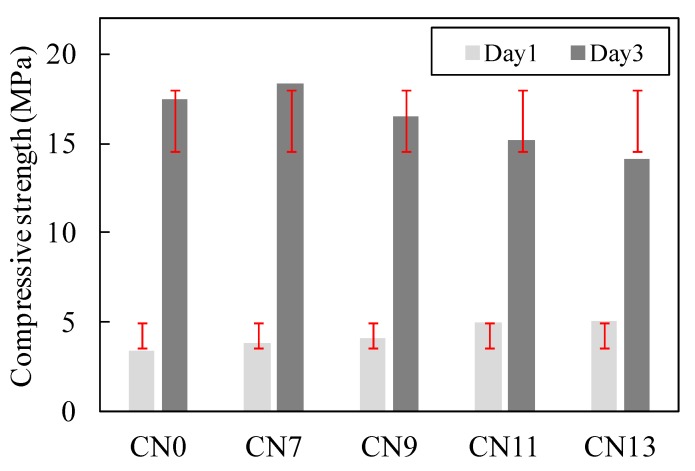
Compressive strength on Days 1 and 3.

**Figure 6:**
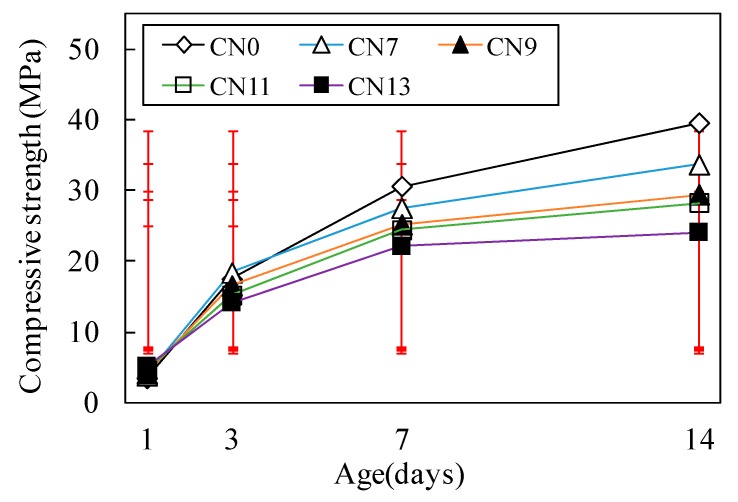
Compressive strength at all ages.

**Figure 7:**
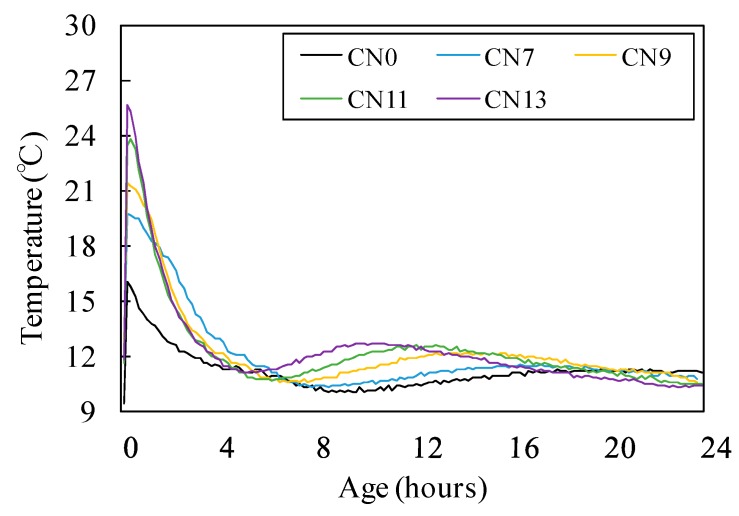
Temperature history (24 h).

Figure 5 and Figure 6 show the result of the compressive strength test for each case where varying amounts of nitrite–nitrate based accelerator (CN) were added. Figure 7 shows the changes in the internal temperature of each mortar specimen from immediately after the mixing process until two hours afterwards. 

Initially, the compressive strength of concrete at Day 1 (early age) was 3.4 MPa for CN0, 3.8 MPa for CN7, 4.1 MPa for CN9, 5.0 MPa for CN11, and 5.1 MPa for CN13, indicating a positive correlation between the amount of CN added and early strength. It is noteworthy that the early strengths of CN11 and CN13, containing large amounts of CN, were 32% and 33% higher, respectively, than that of CN0 (Refer to Figure 5). In addition, as shown in Figure 7, there was a positive correlation between the amount of CN added and the internal temperature of the mortar specimens at 2 h after mixing. In particular, when CN11 and CN13 were added, peak temperatures were reached at around 10 h after the mixing process. This shows that the rate at which their temperatures rose was faster than the temperature rise observed due to the addition of CN0, which reached its peak temperature at around 20 h after the mixing process. According to the existing literature, adding calcium nitrite (CN) to concrete raises the solubility of C_3_S and βC_2_S [9], which in turn promotes the hydration reaction in cement. As a result, hydration products such as C-S-H gel and Ca(OH)_2_ are generated at a faster rate, and this is deemed to have an impact on the heat of hydration occurring in the process (Refer to Figure 7). Thus, based on the above results, it is suggested that adding large amounts of CN results in high strength development at Day 1 (early age). This is attributed to the increase in the absolute amounts of NO_2_^−^ and NO_3_^−^ ions that promote the reaction with C_3_A [12,13], thereby ultimately increasing the amount of nitrite-AFt and nitrate-Aft produced. On the other hand, in the case of CN0, the rate of hydration reaction involving C_3_A, which has an impact on the early strength, as well as C_3_S and C_2_S, is lower than reaction speeds where CN has been added. This, in turn, reduces the rate and amount of hydrate formation. This process is; thus, concluded to be the reason that the early strength of CN0 was lower than that of the other specimens containing CN. 

Figure 5 shows that the compressive strength at Day 3 dropped at a greater rate with increasing amounts of CN. Moreover, as shown in Figure 6, all of the cases in which CN was added displayed a large drop in strength compared to CN0 at Day 7 and Day 14. This is due to the formation of large amounts of needle-type nitrite and nitrate hydrates that exhibit a brittle fracture behavior, resulting from the promotion of reactions between the NO_2_^−^ and NO_3_^−^ ions and C_3_A in the cement [12,13]. This is considered to be the reason for the drop in strength from Day 3 and onwards. Moreover, when nitrite and nitrate hydrates are produced in large amounts, a large quantity of H_2_O in the mixing water is consumed in the process [10,12,13], leading to the possibility that the total amount of C-S-H gel and Ca(OH)_2_ produced from the hydration reaction will be reduced. Meanwhile, CN0 exhibited the general hydration reaction patterns of normal Portland cement, and its temperature curve had a gentler slope compared to that of CN11, as shown in Figure 7. Furthermore, it reached its peak temperature at around 20 h after the end of the mixing process. Based on this, we can conclude that when CN is not added, C_3_S and C_2_S in the cement undergo a normal hydration reaction, without being affected by NO_2_^−^ and NO_3_^−^ ions from the accelerator agent. This, in turn, leads to densification of the cement matrix and is regarded as the reason for the increase in strength from Day 3 onwards. This is in contrast to the strength characteristics observed for other cases where CN was added (Refer to Figure 5 and Figure 6).

### 3.3. Correlation Between Hydration Products and Strength of the Ca(No_2_)_2_·Ca(No_3_)_2_ Concrete by Micro-Analysis

#### 3.3.1. X-Ray Diffraction (XRD)

In order to analyze the relationship between the crystal structure of the nitrite and nitrate hydrates and strength in the cases where the nitrite–nitrate based accelerator (CN) has been added, the hydration products at Day 1 and Day 14 for CN0, which did not contain any CN, and CN13, with 13% CN, were identified by XRD.

**Figure 8:**
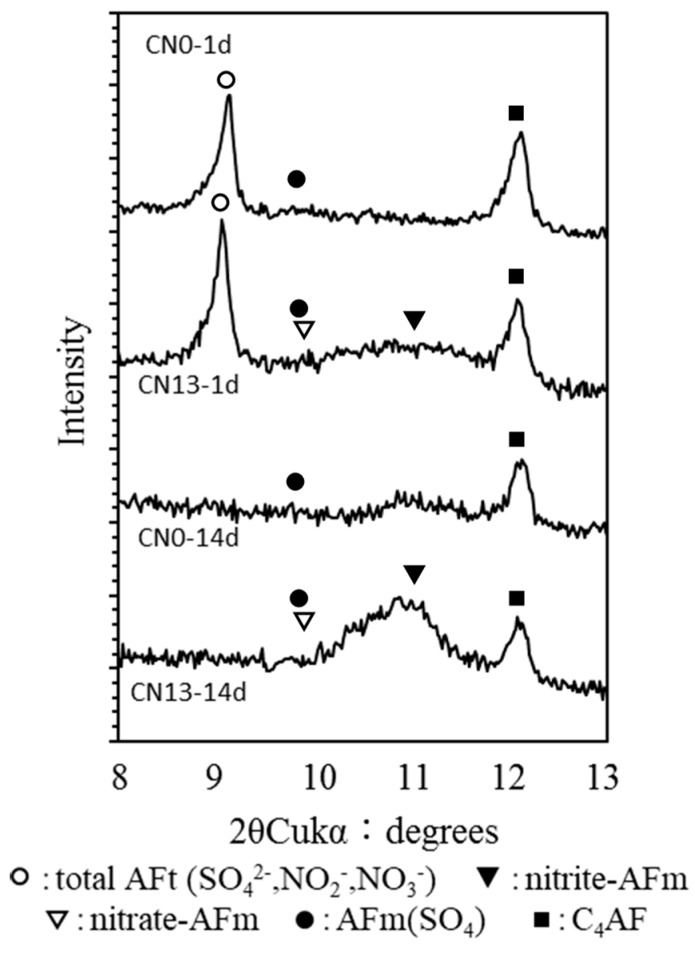
XRD of CN0 and CN13 (Day 1 and Day 14).

The hydrates generated after adding CN contain SO_4_^2−^ ettringite (AFt). They would also include nitrite-Aft and nitrate-Aft resulting from nitrite ions (NO_2_^−^) and nitrate ions (NO_3_^−^). The latter inclusion is caused by CN occupying the areas between the layers of the cement matrix in place of sulfate (SO_4_^2−^), nitrite-AFm(Ca_4_Al_2_(NO_2_)_2_(OH)_12_·4H_2_O), and nitrate-AFm(Ca_4_Al_2_(NO_3_)_2_(OH)_12_·4H_2_O) [13,14,15]. According to previous research, the peak of the formation of nitrite-AFm differs slightly depending on the dryness level, but it typically appears at around 11.04° to 11.23° [14,15]. Therefore, in this study, hydrate identification was carried out between 8° and 13°. Figure 8 shows the XRD analysis results on the calcium-alumina hydrates at Day 1 and Day 14 for CN0 and CN13. 

The XRD results show that at Day 1, ettringite was produced in both CN0 and CN13. However, the peak strength was slightly higher for CN13 than for CN0 due to the influence of the nitrite (NO_2_^−^) and nitrate ions (NO_3_^−^), which may have caused a slight increase in the total AFt in CN13. Further, in the case of CN13, peaks indicating the production of nitrite-AFt and nitrate-AFt, resulting from a rapid reaction between the nitrite ions (NO_2_^−^), nitrate ions (NO_3_^−^), and C_3_A in the cement [12,13], as well as between nitrite-AFm and nitrate-AFm, were observed. Based on these results, it is believed that in the case of CN13 containing a large amount of CN, the matrix of the mortar specimen became densified due to the increased production of SO_4_^2−^ ettringite (AFt) as well as nitrite-AFt, nitrate-AFt, nitrite-AFm, and nitrate-AFm. The result of which was the improvement of early strength.

On the other hand, at Day 14, the ettringite observed on Day 1 was not found in either CN0 or CN13. We conclude that this is because most of the ettringite underwent a change in the crystal structure into mono-sulfate, which resulted in the movement of the peak. It is worthy to note that CN13 showed a pronounced increase in the peaks of nitrite-AFm and nitrate-AFm when compared to the peaks on Day 1. We speculate that this was caused by reactions between nitrite-AFt and nitrate-AFt, which were produced in large amounts at the early age, and Ca(OH)_2_ in the cement over time [9,16]. These reactions caused the transformation of the crystal structure of nitrite-AFt and nitrate-AFt into nitrite-AFm and nitrate-AFm. Based on this, the lower strength of CN13, compared to that of CN0 on Day 3, is ascribed to the relatively smaller production of hydrates, such as C-S-H gel and Ca(OH)_2_, and a subsequent increase in nitrite-AFm and nitrate-AFm. These hydrates, which exhibit a brittle fracture behavior, were produced due to an increase in needle-type nitrite-AFt and nitrate-Aft.

#### 3.3.2. Nuclear Magnetic Resonance (NMR)

**Figure 9:**
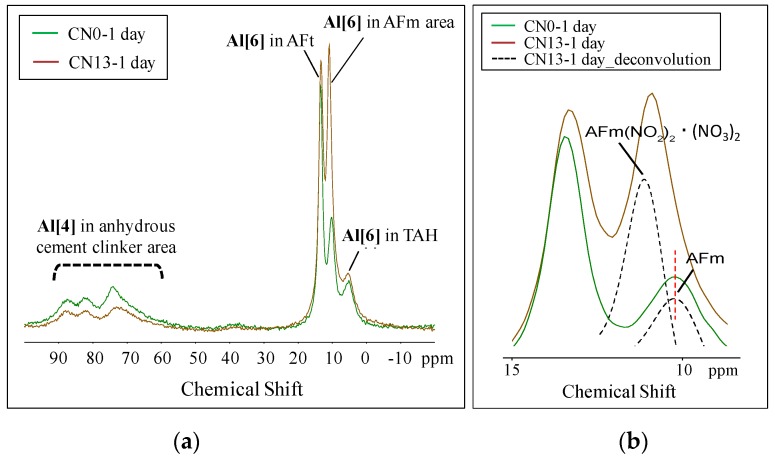
^27^Al NMR spectra of CN0 and CN15 (Day 1). (**a**) Total; (**b**) AFm area.

**Figure 10:**
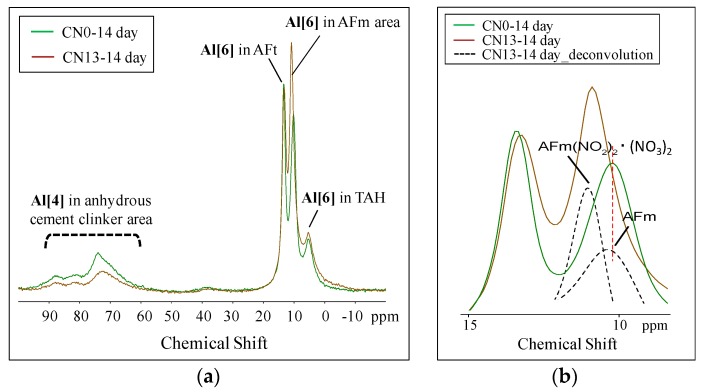
^27^Al NMR spectra of CN0 and CN15 (Day 14). (**a**) Total; (**b**) AFm area.

In order to analyze the relationship between Al hydrates, produced as a result of the reactions between nitrite ions (NO_2_^−^) and nitrate ions (NO_3_^−^) from the nitrite–nitrate based accelerator (CN) and Al_2_O_3_ of C_3_A in the cement, and concrete strength, ^27^Al MAS NMR was used. The measurement results of CN0 and CN13 at Day 1 and Day 14 are shown in Figure 9 and Figure 10. 

Initially, in ^27^Al MAS NMR, a chemical shift of 50–100 ppm for a tetrahedral structure (4 coordination; hereinafter referred to as Al {4}), 30–40 ppm for a pentahedral structure (5 coordination; hereinafter referred to as Al {5}), and 10–20 ppm for an octahedral structure (6 coordination; hereinafter referred to as Al {6}) was observed [17,18,19]. Generally, a large range of resonance from 50 to 80 ppm is known to be the peak for un-hydrated materials with a low degree of crystallinity [20,21,22].

The NMR measurement results show that at Day 1, CN13 had a lower peak height and a smaller peak area at Al {4} (50–80 ppm) in comparison with CN0. Based on this, it was confirmed that adding CN promoted the reaction with C_3_A (Refer to Figure 9a). Further, regarding the peaks in the Al {6} range, that indicate the presence of hydrates produced from the hydration reaction, CN13 showed a peak near 13 ppm, which is known to be indicative of the presence of ettringite (AFt) [17,18], at 9.7 ppm for monosulfate (AFm) [17,18], and a peak for the third aluminate hydrate (TAH) [19]. However, in the case of CN0, while all the peaks in the Al {6} range were detected, the patterns were different. To begin with, in the case of ettringite that has a peak at 13 ppm, the peak area was similar for CN0 and CN13. Firstly, in the case of monosulfate (11–9 ppm), CN13 had a relatively taller and larger peak than CN0, based on which we concluded that the crystal structure of ettringite changed into that of monosulfate on Day 1. This occurred because of the promotion of the reaction with C_3_A in the cement caused by the presence of nitrite ions (NO_2_^−^) and nitrate ions (NO_3_^−^). In addition, with respect to the AFm peak, the center of the peak moved toward a higher ppm in the case of CN13 when compared to the corresponding peak center of CN0. This was due to an overlap between AFm (9.7–10.3 ppm) and the AFm with a different bonding structure detected at 10.7–10.9 ppm, resulting in a broader peak, as shown in Figure 9b. The overlapping peaks can be seen through the results of deconvolution. 

The peak height and area of Al {4} at Day 14 were similar to the patterns exhibited at Day 1 (Refer to Figure 10a). However, as shown in Figure 10b, the Al {6} range, the AFt peak was lower compared to the Day 1 peaks for both CN0 and CN13, whereas the peak area of AFm increased. Further, in the case of CN0, the amount of increase in AFm was slightly higher compared to its value on Day 1. However, relatively speaking, the peak area of CN13 was large, and the differences in the center of the peak, resulting from the presence and absence of CN, were noticeable. In addition, with respect to TAH at 5 ppm, the relative area slightly decreased over time in the case of CN0, and, in contrast, increased over time for CN13. It should be noted that TAH, a phase separate from the amorphous and irregular aluminate hydrate or calcium aluminate hydrate, may form from surface precipitates of C-S-H [19,23].

These results, therefore, confirmed that adding CN promotes the reaction with C_3_A, leading to the possible production of nitrite-AFm and nitrate-AFm in large amounts, in addition to the production of AFm, and to an increase in TAH production. An increase in Al hydrates such as AFm, nitrite-AFm, nitrate-AFm, and TAH at the early age may lower the production of other hydrates such as C-S-H gel and Ca(OH)_2_, and this may be correlated with the decline in strength at the late age.

#### 3.3.3. Scanning Electron Microscope (SEM)

For a comparative analysis of the nitrite (NO_2_^−^) and nitrate (NO_3_^−^) crystals of the hydrates produced as a result of adding the nitrite–nitrate based accelerator (CN), an SEM analysis of the Al hydrates at Day 1 (early age) for CN0 and CN13 was performed. Those results are discussed here with references made to the XRD and NMR results as well. The crystal form of the Al hydrates were first theorized and later validated by making comparisons between the crystal forms and sizes of Al hydrates reported in previous studies and those obtained from this study. 

**Figure 11:**
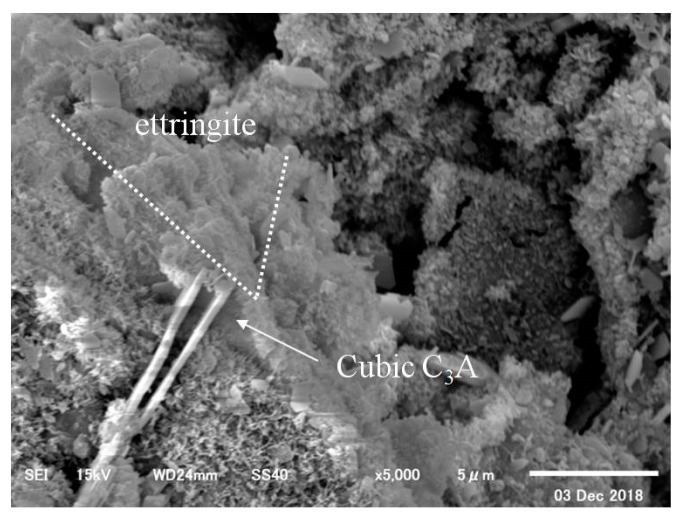
CN0 (×5000)–1.

**Figure 12:**
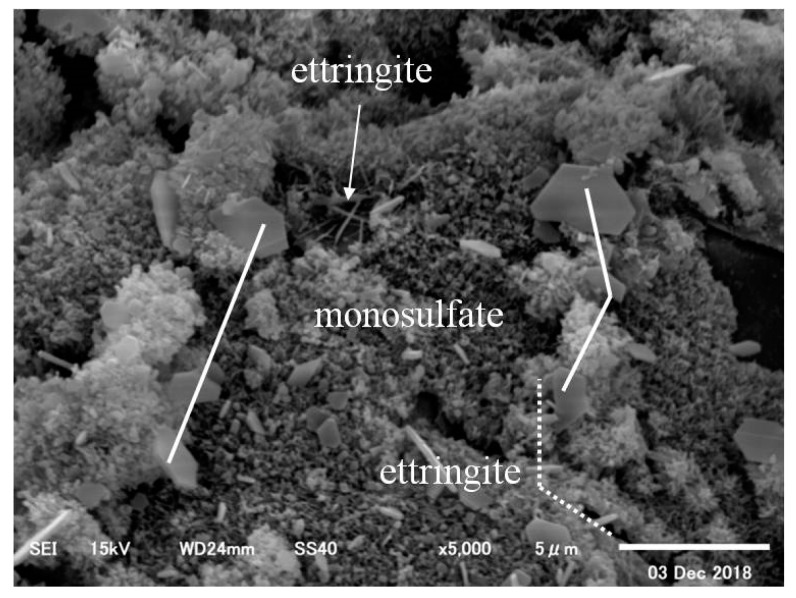
CN0 (×5000)–2.

Initially, in the case of CN0, it was found that sulfate (SO_4_^2−^) ettringite was attached to some parts of the C_3_A surface of the cement, as shown in Figure 11. Further, as shown in Figure 12, sulfate (SO_4_^2−^) ettringite and monosulfate were also observed. 

**Figure 13:**
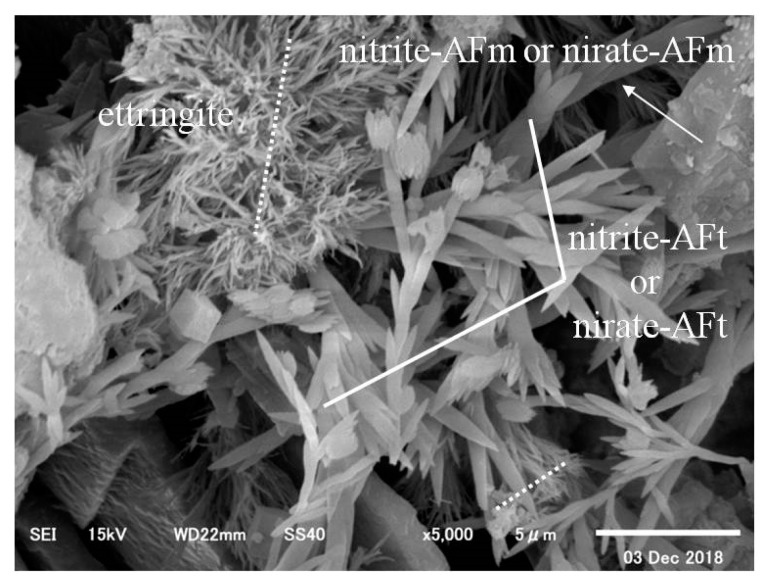
CN13 (×5000)–1.

**Figure 14:**
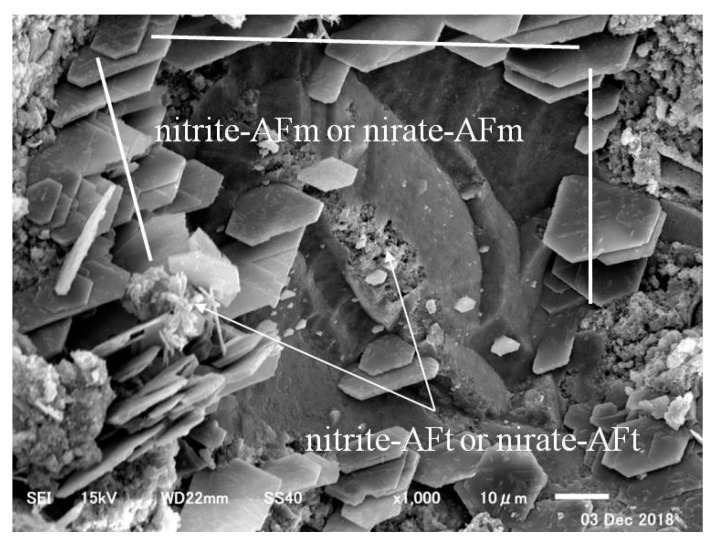
CN13 (×1000)–2.

In the case of CN13, on the other hand, there were large amounts of needle-type nitrite and nitrate hydrates produced from the reactions between the nitrite (NO_2_^−^) and nitrate ions (NO_3_^−^) from the CN and C_3_A in the cement [12,13], in addition to sulfate (SO_4_^2−^) ettringite from normal Portland cement, as shown in Figure 13. Moreover, as shown in Figure 14, in addition to sulfate (SO_4_^2−^) ettringite, nitrite-AFm and nitrate-AFm were widely observed because they were produced from the reactions between nitrite-AFt and nitrate-AFt and Ca(OH)_2_ in the cement [9,16].

## 4. Conclusions

In this study, diverse physicochemical reviews were performed to clarify the relationship between the hydrate formation behavior and strength development at early and later ages of concrete by varying the amount of nitrite–nitrate based accelerator, which is an anti-freezing agent. The results of the study may be summarized as follows:

(1) With regards to fluidity, an increase in CN raised the rate of reaction between the nitrite (NO_2_^−^) and nitrate ions (NO_3_^−^), which are a part of the CN, and Al_2_O_3_ of C_3_A in the cement, which in turn caused the heat of hydration to increase. This consequently raised the internal temperature of the mortar and reduced its fluidity;
(2) Concerning strength development over time, an increase in CN raised the rate and amount at which nitrite-AFt and nitrate-AFt were formed, and this in turn increased the amount of nitrite-AFm and nitrate-AFm. This increased strength at the early age; however, due to the production of large amounts of hydrates with vulnerable crystal structures, we conclude that the strength would be reduced at the later ages;
(3) The XRD and NMR results showed that the cases where CN was added resulted in a larger peak height and area of ettringite, that is an Al hydrate, than the case without CN, due to the influence of nitrite (NO_2_^−^) and nitrate ions (NO_3_^−^) at the early age. Nitrite-AFm and nitrate-AFm peaks were also observed. Further, at the late ages, the needle-type nitrite-AFt and nitrate-Aft, which were produced in large amounts at the early age, reacted with Ca(OH)_2_ in the cement over time, resulting in the transformation of their crystal structure into those of nitrite-AFm and nitrate-AFm;
(4) The NMR results showed that an increase in Al hydrates such as AFm, nitrite-AFm, nitrate-AFm, and TAH, resulting from the addition of the CN, caused a relative decrease in the production of other hydrates such as C-S-H gel and Ca(OH)_2_, thereby lowering strength at mid and late ages.
(5) The SEM examination results showed that adding CN to concrete as an accelerator resulted in the production of a large amount of needle-type nitrite and nitrate hydrates. This is due to the reactions between the nitrite (NO_2_^−^) and nitrate ions (NO_3_^−^) from the CN and C_3_A in the cement, in addition to the production of sulfate (SO_4_^2−^) ettringite from the reaction of normal Portland cement. We also observed the presence of nitrite-AFm and nitrate-AFm produced from the reactions between nitrite-AFt and nitrate-AFt and Ca(OH)_2_ in the cement.

## Figures and Tables

**Table 1:** Properties of the materials used.

Materials (Code)	Properties
Cement (C)	Normal Portland cement, Density: 3.16 g/cm^3^
Fine aggregate (S)	No. 5 silica sand, Absolute dry density: 2.61 g/cm^3^, Water absorption: 0.26%, Fineness modulus: 2.16
Anti-freezing agent (CN)	nitrite–nitrate based accelerator = Calcium nitrite (Ca(NO_2_)_2_); Calcium nitrate (Ca(NO_3_)_2_)

Note: CN: Anti-freezing agent = nitrite + nitrate-based accelerator (Ca(NO_2_)_2_ + Ca(NO_3_)_2_)

**Table 2:** Properties of the anti-freezing agent.

Code	Component	Component Ratio	pH	Specific Gravity
CN	Ca(NO_2_)_2_	23.02%	9.3	1.43
	Ca(NO_3_)_2_	22.81%		

**Table 3:** Proportions of the mortar mix.

Type	W/C (%)	S/C	Unit Content (kg/m^3^)			Anti-Freezing Agent (C × %)
			W	C	S	CN
CN0	50	2.0	315	631	1262	0
CN7						7
CN9						9
CN11						11
CN13						13

Note: W/C: water cement ratio; S/C: sand cement ratio; CN0: Mixing amount of anti-freezing agent = 0%; CN7: Mixing amount of anti-freezing agent = 7%; CN9: Mixing amount of anti-freezing agent = 9%; CN11: Mixing amount of anti-freezing agent = 11%; CN13: Mixing amount of anti-freezing agent = 13%

**Table 4:** Experimental conditions and evaluation method.

Temperature Condition	Experimental Period	Subject and Method of Evaluation	
		Physical Properties	Chemical Properties
10 °C	Casting—14 days	-Flow test -Compressive strength -Temperature history	-XRD -NMR -SEM

Note: Physical properties: All cases; Flow test: Immediately after placement; Compressive strength: Days 1, 3, 7, and 14; Temperature history: Casting—14 days; Chemical properties: XRD, NMR: CN0, CN13, and Days 1 and 14; SEM: CN0, CN13 and Day 1 only.
